# Clustering recovered 18650 lithium-ion cells to improve homogeneity in second-life battery pack construction

**DOI:** 10.1371/journal.pone.0353394

**Published:** 2026-07-16

**Authors:** Victor Olivero-Ortiz, Ingrid Oliveros Pantoja, Jean Mendoza Polo, Carlos Robles-Algarín, Lácides Ripoll Solano

**Affiliations:** 1 Facultad de Ingeniería, Universidad del Magdalena, Santa Marta, Magdalena, Colombia; 2 Departamento de Ingeniería Eléctrica y Electrónica, Universidad del Norte, Barranquilla, Atlántico, Colombia; SVM Arts, Science and Commerce College, INDIA

## Abstract

Second-life use of lithium-ion batteries depends on assembling packs from recovered cells that behave as consistently as possible; however, recovered cells are often heterogeneous because of mixed models and different aging histories. This study aimed to improve pack-level homogeneity by clustering recovered cylindrical 18650 cells using laboratory-measured electrical variables and validating the approach through the construction and testing of a second-life pack. Cells were extracted from discarded laptop batteries, screened using safety and basic performance criteria, and characterized under a standardized charge–discharge protocol. For each retained cell, discharged capacity and internal resistance were measured, and health indicators were computed from nominal specifications and experimental results. Correlation analysis was used to identify the most informative variables associated with health status and to avoid redundant predictors prior to clustering. Clustering performance was evaluated across different numbers of groups using a cohesion–separation index, and two iterative selection algorithms were applied to select non-overlapping, highly homogeneous subsets of cells for series–parallel pack assembly. Using the same pack topology, the refined algorithm produced a second-life pack with higher usable capacity (8.98 Ah versus 8.72 Ah), lower equivalent internal resistance (76.38 mΩ versus 78.90 mΩ), and higher estimated maximum power (716.97 W versus 694.08 W) compared with the baseline algorithm. Overall, the results show that clustering guided by discharged capacity and internal resistance, combined with iterative refinement, enables the construction of more homogeneous and electrically consistent second-life packs from heterogeneous recovered cells.

## Introduction

The wide range of applications of lithium-ion batteries has made them one of the primary drivers of the ongoing energy transition, although their early use was mainly limited to consumer electronics. Lithium-ion batteries can be utilized in two distinct phases: first life and second life. During their first life, cells, packs, and complete battery systems are used in electromobility, portable electronic devices, and, more recently, energy storage systems integrated with renewable energy sources. In their second life, these batteries are dismantled, tested, classified, grouped, and reassembled for new applications, typically those with lower energy or power demands [1]. Each lithium-ion battery cell stores energy as chemical energy through a structure composed of two electrodes, an anode and a cathode, separated by an electrolyte. These components enable reversible redox reactions that allow the cell to be charged and discharged. According to John B. Goodenough, the critical parameters of a rechargeable battery include safety, energy density (defined as the amount of energy that can be stored for a given input and delivered at a given output), cycle life and overall lifetime, storage efficiency, and manufacturing cost [2].

These features have positioned lithium-ion batteries as one of the most advanced energy storage technologies, with substantial gains in energy performance. Early commercial models offered specific energy of about 80 Wh/kg, whereas current high-energy-density variants used in power-oriented applications reach 240–250 Wh/kg. Even higher values, from 260 to 295 Wh/kg, are reported for batteries used in computing, communication, and consumer electronics devices, commonly referred to as 3C devices [3]. Lithium-ion battery production has also grown rapidly, rising from 210 GWh/year in 2005–1,000 GWh/year by 2025, with projections of up to 1,500 GWh/year by 2030 [4]. From 2005 to 2030, this corresponds to an overall increase of approximately 614%. By 2030, the portable electronics sector alone is expected to account for 50–60 GWh of global battery demand (4). This sustained growth is driven by increasing demand for energy storage, largely fueled by electric vehicles, photovoltaic systems, and other applications aligned with the global energy transition [5].

The growing demand for and production of lithium-ion batteries also raise serious concerns about the increasing number of batteries reaching end of life (EOL), which could lead to a substantial rise in waste that must be properly managed. Mrozik examined the environmental impacts, pollution, and risks to human life and health that can arise when these batteries are not disposed of correctly. For example, landfilling has been reported as the most common practice, although recycling is becoming increasingly prevalent [6]. Given the uncertainty surrounding the final disposition of lithium-ion batteries, whether due to the absence of regulatory frameworks or challenges in enforcing them, circular economy approaches have gained momentum, with an emphasis on recycling and second-life applications. A Delphi panel reported in [7] outlined the value chain for reusing electric vehicle batteries and concluded that characterization and classification are key factors in determining whether a battery is suitable for second-life use or should instead be processed as a source of raw materials. Regarding the most appropriate circular business model (CBM), the experts proposed a combined strategy of remanufacturing, reuse, recycling, and waste management to maximize value recovery from used lithium-ion batteries.

Implementing a lithium-ion battery reuse model typically begins with a physical stage that focuses on disassembling and dismantling the battery system. This process enables the identification of key components, including modules, packs, cells, battery management systems (BMS), sensors, and electrical connections. Reuse strategies can be applied across a wide range of products, from consumer electronics to electric vehicles. For example, several studies have examined second-life pathways for laptop battery cells and reported that some components retain more than 70% of their original capacity [8].

From a technological standpoint, one of the fundamental steps in assembling new batteries from reused components is classifying and grouping used cells. Among the most commonly referenced cell types in this context are cylindrical 18650 cells, which are widely used in electronic devices and defined by standardized dimensions of 18 mm in diameter and 65 mm in height. However, newer formats such as 21700 and 26650 cells, which offer higher capacities and improved volumetric efficiency, are increasingly being adopted. Beyond form factor, cells are also distinguished by their internal chemistry, which governs their electrical and energy-related characteristics. For example, LCO (LiCoO2) and NMC (LiNiMnCoO2) cells typically have a nominal voltage of 3.7 V, with discharge voltages around 3.0 V. LCO cells commonly achieve specific energies of approximately 200–250 Wh/kg, whereas NMC cells typically range from 150 to 220 Wh/kg. These variations underscore the importance of characterizing, classifying, and grouping cells not only using basic electrical parameters, but also accounting for usage history, state of health (SoH), and charge and discharge behavior.

Machine learning techniques have emerged as effective tools for improving the grouping and assembly of battery packs that are as homogeneous as possible, particularly when first-life data are unavailable. Clustering, an unsupervised learning approach, is used to uncover patterns in unlabeled data. In clustering, the goal is to partition a dataset into subsets (clusters) so that the results are interpretable and can be evaluated using validity indices that favor high within-cluster similarity and low between-cluster similarity [9]. Common clustering methods include k-means, fuzzy clustering, hierarchical clustering, kernel k-means, spatial clustering, spectral clustering, and self-organizing maps (SOMs) [10]. Clustering performance is often assessed using similarity metrics and validity indices such as Euclidean distance, the silhouette coefficient, the Dunn index, the Davies-Bouldin index, and the Calinski-Harabasz index, along with density-based, topology-based, and information-theoretic measures such as the V-measure. In contrast, more advanced approaches use parameter-tuned models that explicitly balance within-cluster cohesion and between-cluster separation to produce an overall clustering score [11].

The application of clustering algorithms to group lithium-ion cells has become a practical strategy for reducing inhomogeneity during battery pack construction and mitigating its effects on lifespan, performance, and safety in both new and second-life batteries. Although a wide range of features has been reported, several appear consistently across the literature. In [12], 16 variables were used as an initial feature set, followed by feature selection using Pearson correlation and a second stage based on multiple regression. After applying k-means, the authors identified five clusters characterized by high discharge capacity and low voltage deviation among cells. Several studies also highlight clustering, particularly k-means, together with correlation analysis among features, as an effective approach for cell grouping [13,14]. Other works compare multiple clustering methods, including DBSCAN, improved k-means variants, affinity propagation, and newer approaches such as traversal optimization [15,16]. In addition, the authors in [16] used principal component analysis (PCA) to reduce dimensionality and capture most of the variance across features such as charge capacity, discharge capacity, charge energy, discharge energy, Ah efficiency, Wh efficiency, and voltage inconsistency.

Second-life batteries and the grouping of cells suitable for reuse have also been investigated using classification and clustering approaches informed by degradation and aging mechanisms. Using methods such as fuzzy clustering and features derived from loss of active material (LAM) and incremental capacity analysis (ICA), researchers have characterized cells for applications including stationary energy storage and peak-load shifting [17]. However, when the available inputs come primarily from experimental testing of used cells, such as capacity, internal resistance, and remaining useful life (RUL), alternative approaches have been proposed that adapt support vector methods. In the clustering context, these are often referred to as support vector clustering (SVC) [18].

Zhu et al. [19] developed a fast and efficient methodology to estimate and regroup retired lithium-ion cells to enable reuse across multiple application scenarios. Their approach combines a support vector regression (SVR) model optimized with particle swarm optimization (PSO) and reports capacity estimation errors below 0.3%. In addition, they implemented weighted k-means clustering to improve voltage consistency, allowing cells to be matched to specific energy or power requirements. Lai et al. [20] proposed an efficient method for rapid classification and regrouping for second-life applications using an improved k-means algorithm tailored to different cascaded utilization scenarios. Their method reported capacity estimation errors below 3% and improved the electrical consistency of the regrouped system. Ran et al. [21], focusing on short pulse testing, used an enhanced bisecting k-means algorithm integrated into a high-throughput intelligent sorting machine, achieving up to 88% accuracy relative to full charge and discharge testing. Liu et al. [22] used Pearson correlation and grid search to identify features strongly correlated with capacity, thereby improving clustering performance. They also proposed a fusion mechanism that combines improved k-means, fuzzy c-means, hierarchical clustering, and spectral clustering. Experiments using 97 real cells and up to 600 simulated cells reported classification accuracy above 95%, supporting the method’s effectiveness across diverse operating conditions. However, there is no consensus on which clustering strategy and validity indices best translate into improved pack-level performance when first-life data are unavailable, particularly in studies that move beyond regrouping to pack assembly and experimental validation.

Despite these advances, most existing approaches stop at the clustering stage and do not validate that the resulting groups translate into measurable pack-level improvements. Piombo et al. [23] recently argued that conventional clustering alone is insufficient for application-specific optimization, proposing a combinatorial approach that trades scalability for pack-level performance. The present work addresses this gap with three specific contributions: (i) a complete, experimentally validated workflow that connects cell characterization, clustering-based selection, and physical pack assembly in a single pipeline; (ii) two iterative selection algorithms — one Silhouette-driven (V1) and one with hierarchical refinement (V2) — formalized as reproducible pseudocode with published source code; and (iii) a statistical comparison framework (random-selection baseline, bootstrap confidence intervals, effect-size analysis) that quantifies the advantage of clustering-guided assembly over naïve selection. The methodological contribution lies in demonstrating that even with consumer-grade instrumentation and heterogeneous recovered cells, a structured data-driven approach produces packs that are statistically superior to random assembly.

## Materials and methods

This section describes the materials, experimental procedures, and analytical methods used to evaluate and cluster second-life lithium-ion cells recovered from laptop battery packs. The goal was to build homogeneous battery modules by applying and comparing clustering algorithms using key electrical characteristics measured through standardized tests. The workflow included battery pack disassembly, individual cell testing, data preprocessing, feature selection, and the implementation of both baseline and advanced clustering techniques. Overall, the methodology was designed to reflect a practical cell-to-package process for second-life battery repurposing.

### Variable selection for clustering using correlation analysis

Because the ultimate goal is to build second-life battery modules, only variables that provide relevant information about the cells’ state of health (SoH) were selected. Accordingly, both feature selection and clustering were based on parameters that reflect cell degradation, which helps form homogeneous groups in terms of performance and durability. To identify the most relevant variables, correlation analysis was performed using Pearson’s correlation coefficient, which quantifies the linear relationship between two variables. The coefficient is defined as:


$$\begin{array}{c} =\frac{cov(x,y)}{\sigma_{x}\sigma_{y}} \end{array}$$ (1)

Where *r* is Pearson’s correlation coefficient, *cov(x,y)* is the covariance between variables *x* and *y*, and *σ*_*x*_ and *σ*_*y*_ are the standard deviations of *x* and *y*, respectively. In its expanded form, Pearson’s correlation coefficient is given by:


$$\begin{array}{c} =\frac{\sum_{i=\text{1}}^{n}(x_{i}-\overset{―}{x})(y_{i}-\overset{―}{y})}{\sqrt{\sum_{i=\text{1}}^{n}(x_{i}-\overset{―}{x}{)}^{2}}\sqrt{\sum_{i=\text{1}}^{n}(y_{i}-\overset{―}{y}{)}^{2}}} \end{array}$$ (2)

Where $\overset{―}{x}$ and $\overset{―}{y}$ are the sample means of *x*_*i*_ and *y*_*i*_, respectively, and *n* is the total number of observations.

For the clustering process, two target variables were considered, labeled in the dataset as SoH and Adjusted SoH. Pearson’s correlation coefficient was computed between each remaining variable (excluding the target variables) and both SoH and Adjusted SoH. Variables with an absolute correlation coefficient ∣*r*∣ ≥ 0.3 were retained, indicating a meaningful association with the cells’ degradation state.

Additionally, redundant variables were excluded. Specifically, when two non-target variables showed a high mutual correlation (∣*r*∣ > 0.5), only the variable with the stronger correlation with the target variables (SoH and Adjusted SoH) was retained. This approach helps ensure that the final feature set used for clustering captures distinct and relevant information, supporting the formation of highly homogeneous groups in terms of cell health.

### Methodology for cell selection and second-life battery pack assembly

Recovered lithium-ion cells from laptop battery packs were first characterized to establish their electrical and physical condition prior to regrouping. For each cell, the following attributes were obtained from laboratory testing: state of health (SoH) and adjusted SoH, average internal resistance, remaining capacity, discharge time, initial and final voltage, and weight. Electrical variables were obtained from standardized charge and discharge testing, while physical attributes were obtained through direct measurements. This dataset served as the basis for the clustering, screening, and final selection steps.

An initial clustering stage was then applied to identify groups of cells with similar characteristics. In addition to forming preliminary groups, this stage supported the removal of unsuitable cells by discarding clusters whose average values for critical parameters fell outside predefined acceptance thresholds, such as internal resistance exceeding 150% of the nominal value or SoH below 70% relative to a new cell. This screening step helps improve the safety and reliability of the repurposed module or pack by reducing the likelihood of large imbalances among cells.

Final cell selection was performed using an iterative clustering-based procedure designed to maximize homogeneity in the pack configuration. The number of clusters, *k*, was varied from the minimum required by the target pack design, defined by *P* (the number of parallel groups), up to a maximum value constrained by the dataset size. For each value of *k*, a clustering algorithm was applied to generate a partition of the dataset. The specific technique is described in the subsequent section and included methods such as k-means and Gaussian mixture models (GMM) initialized with k-means. Each partition was evaluated using the global silhouette index together with individual silhouette scores for each cell. Clusters that satisfied the minimum size requirement were retained, and the *P* clusters with the highest internal homogeneity were selected to represent the parallel groups. Within each selected cluster, cells were ranked by their individual silhouette scores, and the top *S* cells were selected, where *S* is the number of cells required in series for each group. This procedure yields *P* highly homogeneous subsets of size *S*, which define the final set of cells used for assembly.

**Fig 1:**
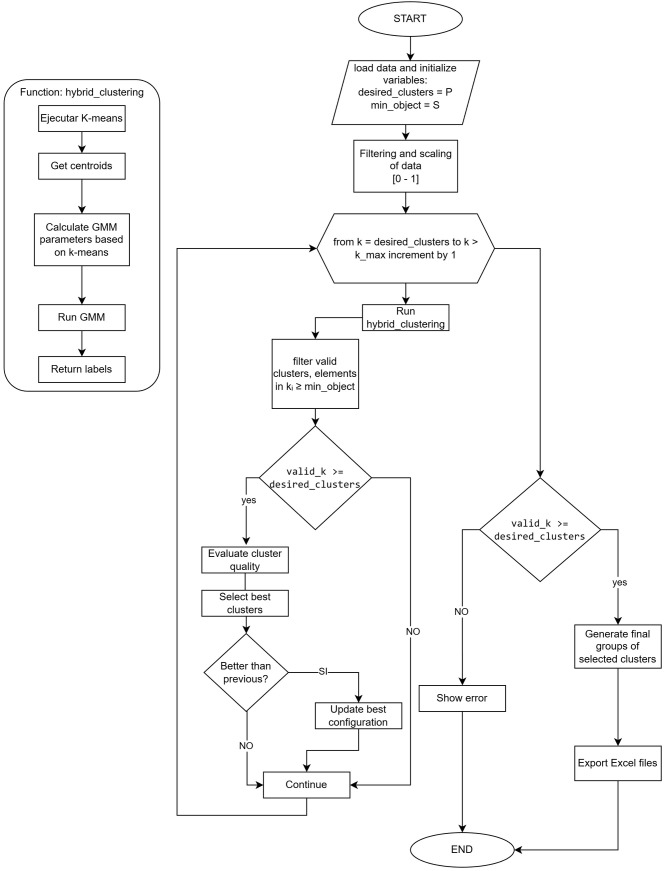
Flowchart of the iterative clustering-based cell selection and second-life battery pack assembly workflow.

Fig 1 summarizes the iterative clustering-based workflow, including partition exploration, clustering, silhouette-based evaluation, and the final selection of the most homogeneous cells for each parallel group. Based on the selected subsets, the battery pack was assembled as *S* cells in series across *P* parallel groups (*S* × *P*). This assembly strategy aims to improve parameter consistency across groups, reduce imbalance, and support stable performance and extended service life during second-life operation. The two selection algorithms are formalized in Table 1 and Table 2 (Table 3 lists all hyperparameters). Version 1 performs an exhaustive search over k, evaluating GMM partitions initialized from K-means centroids and selecting the p most cohesive clusters based on their mean per-cell Silhouette score. Within each selected cluster, the top s cells by individual Silhouette score form one series string. Version 2 extends this procedure with a hierarchical refinement step: clusters exceeding 2s cells are subdivided by a second-level GMM, and subgroups that improve local cohesion are added to a candidate pool. The final pack is assembled by greedily selecting p non-overlapping groups from this pool. Both algorithms use *n*_init_ = 50 to ensure deterministic initialization (see Seed-sensitivity analysis). Source code implementing both algorithms is available in the public repository cited in the Data Availability statement.

**Table 1:** Algorithm 1: Cell selection algorithm V1 (Silhouette-driven).

Algorithm 1: Selection Algorithm V1 (Silhouette-driven)	
	**Input:** X ∈ ℝ^n × d^ (clustered cell features), s (cells per series string), p (number of parallel groups), k_**min**_ = p, k_**max**_ = ⌊n/s⌋
	**Output:** S* ⊆ {1, …, n} with |S*| = s × p
1	best_sil ← −∞
2	**for** k = k_**min**_ **to** k_**max**_ **do**
3	labels ← GMM(X, k, init = KMeans(X,k,n_init = 50,rs = 42))
4	sil_global ← SilhouetteScore(X, labels)
5	sil_i ← SilhouetteSamples(X, labels) // per-cell scores
6	C ← {c: |cluster c| ≥ s} // eligible clusters
7	**if** |C| < p **then** continue
8	Sort C by mean(sil_i[c]) descending
9	G ← top p clusters from C
10	S ← ∅
11	**for each** g ∈ G **do**
12	cells_g ← indices in cluster g, sorted by sil_i descending
13	S ← S ∪ top-s(cells_g)
14	**if** sil_global > best_sil **then**
15	best_sil ← sil_global; S* ← S
16	**return** S*

**Table 2:** Algorithm 2: Cell selection algorithm V2 (Hierarchical refinement).

Algorithm 2: Selection Algorithm V2 (Hierarchical refinement)	
	**Input:** X, s, p, k_**min**_, k_**max**_, k_**sub_max**_
	**Output:** S* ⊆ {1, …, n} with |S*| = s × p
1	labels_0 ← GMM(X, k_**best**_, init=KMeans) // from V1 best partition
2	pool ← ∅ // candidate subgroup pool
3	**for each** cluster c in labels_0 **do**
4	n_**c**_ ← |cluster c|
5	**if** n_**c**_ ≥ 2·s **then** // large enough to subdivide
6	X_**c**_ ← X[cluster c]
7	**for** k_**sub**_ = 2 **to** min(k_**sub_max**_, ⌊n_**c**_/s⌋) **do**
8	sub_labels ← GMM(X_**c**_, k_**sub**_, init = KMeans(n_init = 50))
9	**for each** subgroup sg **do**
10	**if** |sg| ≥ s and mean_sil(sg)> sil(c) **then**
11	pool ← pool ∪ {sg}
12	**else**
13	pool ← pool ∪ {c} // keep original cluster as candidate
14	// Select best non-overlapping groups
15	Sort pool by mean silhouette descending
16	G* ← greedy_select(pool, p, non_overlapping = True)
17	S* ← ∅
18	**for each** g ∈ G* **do**
19	S* ← S* ∪ top-s(g, by silhouette)
20	**return** S*

**Table 3:** Hyperparameter configuration used in the proposed clustering framework.

Parameter	Symbol	Value	Justification
Cells in series	*s*	4	Target pack topology (4s6p)
Parallel groups	*p*	6	Target pack topology (4s6p)
Initial clusters	*k*	6	Maximizes silhouette score
K-means initializations	*n*_*init*_	50	Stability analysis
K-means random state	*random_state*	42	Reproducibility
K-means maximum iterations	*max*_*iter*_	300	scikit-learn default
K-means tolerance	*tol*	10 ⁻ ⁴	scikit-learn default
GMM covariance type	*cov*_*type*_	full	Better fit for non-spherical clusters
GMM initialization	*init*	K-means centroids	Warm start for convergence
Feature scaling	*scaler*	MinMaxScaler [0,1]	Equal weighting of features
Resistance filter	—	R ≤ 400 mΩ	Excludes non-functional cells
Minimum cluster size	—	≥ *s* = 4	Pack topology constraint
Sub-clustering limit	*k_sub*_max_	9	Empirical selection based on cluster size
Software environment	Python	3.11	scikit-learn 1.4, NumPy 1.26, pandas 2.2

### Clustering algorithms and evaluation metric

Two complementary clustering strategies were compared to maximize homogeneity during cell regrouping and to support the final assembly of the second-life battery pack. The first was k-means, which is computationally simple and typically produces convex partitions. The second was a Gaussian mixture model (GMM) initialized from the k-means solution, which fits a probabilistic model and can represent clusters with more flexible shapes. Clustering quality was assessed using the silhouette index, which captures within-cluster cohesion and between-cluster separation. For each algorithm and each tested number of clusters, the average silhouette score was computed, and the configuration with the highest score was selected. This comparison evaluated whether the added modeling flexibility of GMM provided a meaningful improvement over k-means before applying the iterative subclustering procedure and assembling the battery pack.

#### Reproducibility and seed-sensitivity protocol.

Because K-means is sensitive to centroid initialization, every K-means run reported in this work was executed with *n*_*init*_ = 50 (50 independent k-means++ initializations per fit, retaining the partition with the lowest within-cluster inertia), *max*_*iter*_ = 300, and tol = 10 ⁻ ⁴. One cell (COD 30−1; R = 418 mΩ, C = 0.038 Ah) was excluded prior to clustering because its resistance exceeded the 400 mΩ threshold and its near-zero capacity indicated a non-functional cell, leaving 91 cells for all clustering analyses. The Gaussian mixture model was initialized from the resulting K-means centroids. To verify reproducibility, we re-ran the full pipeline with 100 distinct *random_state* values (0–99) at k = 6. Under the adopted protocol (*n*_*init*_ = 50), the Silhouette index varied within 0.466 ± 0.001 (min = 0.465, max = 0.472) and the pairwise ARI exceeded 0.94 in all cases (mean ARI = 0.990), confirming that the partition is essentially deterministic. For comparison, with *n*_*init*_ = 1 the Silhouette range widened to [0.337, 0.472] (std = 0.029) and the mean pairwise ARI dropped to 0.77, illustrating the importance of the multi-start strategy. Complete numerical results are provided in S1 Table and S1 Fig. All analyses used Python 3.11, scikit-learn 1.4, NumPy 1.26, and pandas 2.2; source code and dataset are released in the repository indicated in the Data Availability statement.

K-means was selected as the baseline clustering method because it is a deterministic partitioning algorithm that divides a dataset into *K* clusters, each represented by a centroid *μ*_*k*_ that reflects the central location of the points assigned to that cluster. Unlike probabilistic approaches, k-means assigns each observation to exactly one cluster, which is commonly referred to as hard clustering. Before applying the algorithm, all variables were normalized to place features on a common scale and prevent any single variable from dominating distance calculations. Min–max normalization was used to map each feature to the [0,1] range:


$$\begin{array}{c} x_{i,norm}=\frac{x_{i}-min(x_{i})}{max\left(x_{i}\right)-min\left(x_{i}\right)} \end{array}$$ (3)

Where *x*_*i*_ is the original value of a given feature and *x*_*i,norm*_ is the corresponding normalized value. This transformation ensures that each feature contributes comparably to Euclidean distance computations.

K-means seeks a partition that minimizes the within-cluster sum of squared distances, commonly referred to as the sum of squared errors (SSE) or inertia. This objective function is defined as:


$$\begin{array}{c} SSE=\;J=\sum_{k=\text{1}}^{K}\sum_{x\epsilon C_{k}}{\left\|x-u_{k}\right\|}^{\text{2}} \end{array}$$ (4)

Where *C*_*k*_ denotes the set of points assigned to cluster *k*, *μ*_*k*_ is the centroid of cluster *k*, and ∥*x* − *μ*_*k*_∥^2^ is the squared Euclidean distance between point *x* and its assigned centroid. The goal is to find the partition {*C*_*1*_,*C*_*2*_,…,*C*_*K*_} and the centroids {*μ*_*1*_,*μ*_*2*_,…,*μ*_*K*_} that minimize *J*. By setting the partial derivative of *J* with respect to *μ*_*k*_ to zero, the optimal centroid condition is obtained:


$$\begin{array}{c} \frac{\partial J}{\partial \mu_{k}}=\sum_{x\epsilon C_{k}}\text{2}(x-u_{k})=\text{0} \end{array}$$ (5)

Solving Eq. (5) shows that the centroid is the arithmetic mean of the points assigned to cluster *k*:


$$\begin{array}{c} \mu_{k}=\frac{\text{1}}{\left|C_{k}\right|}\sum_{x\epsilon C_{k}}x_{i} \end{array}$$ (6)

This update ensures that *μ*_*k*_ represents the central location of all points within *C*_*k*_ under the squared Euclidean distance criterion.

K-means proceeds iteratively through initialization, assignment, and update steps. First, *K* centroids are initialized, either at random or using a heuristic initialization. Next, each observation *x*_*i*_ is assigned to the cluster whose centroid *μ*_*k*_ minimizes the squared Euclidean distance:


$$\begin{array}{c} C_{k}=\left\{x_{i}:∥x_{i}-\mu_{k}{∥}^{\text{2}}\;\leq \;∥x_{i}-\mu_{m}{∥}^{\text{2}}\forall \;m\neq k\right\} \end{array}$$ (7)

Gaussian mixture models (GMMs) were also evaluated because they provide a probabilistic clustering framework in which the data are modeled as a mixture of K multivariate Gaussian components. Unlike k-means, which performs hard assignments, GMMs allow soft assignments in which each observation can belong to multiple components with different probabilities. Each component k is parameterized by a mean vector μk, a covariance matrix Σk, and a mixing coefficient πk. Model parameters are estimated by maximizing the data log-likelihood via the expectation–maximization (EM) algorithm, which alternates between computing posterior assignment probabilities (E-step) and updating component parameters (M-step) until convergence [24]. Both full and spherical covariance structures were tested. The complete mathematical formulation is provided in S1 Appendix.

The silhouette index was used to quantify clustering quality in terms of within-cluster cohesion and between-cluster separation. For each observation *i*, the silhouette score is defined as:


$$\begin{array}{c} s(i)=\frac{b(i)-a(i)}{max\left\{a(i),b(i)\right\}} \end{array}$$ (8)

Where *a(i)* is the average distance between *i* and all other points in the same cluster, and *b(i)* is the minimum average distance between *i* and points in any other cluster. The mean silhouette score across all observations was computed for each tested *k* and used to select the clustering configuration. Individual silhouette scores were additionally used to rank cells within retained clusters during the final selection stage.

### Experimentation with 18650 cells, data extraction, and dataset construction

The construction of second-life modules and battery packs requires lithium-ion cells that have completed their first-life use in consumer electronics or electric mobility. Once these cells no longer meet their original specifications or performance requirements, they may be repurposed for applications that typically impose lower power or energy demands.

**Fig 2:**
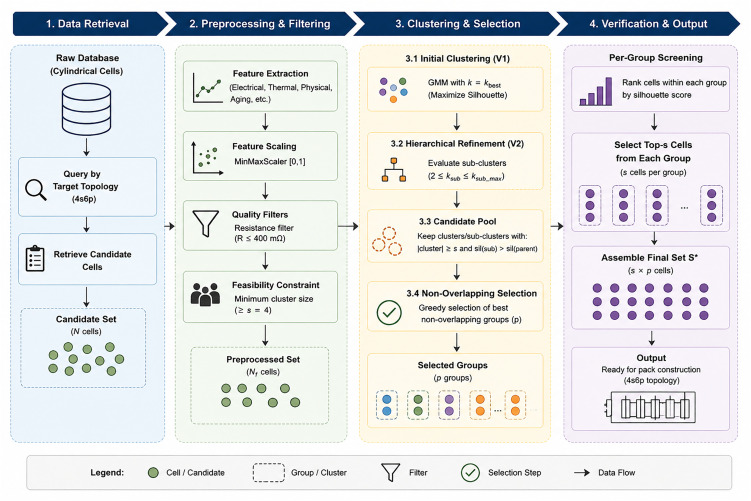
Cell recovery and screening workflow for the 18650 cells used in this study.

A total of 123 cylindrical 18650 cells were recovered, primarily from laptop battery packs from multiple manufacturers, then labeled and tested to support second-life module assembly. Fig 2 summarizes the recovery and screening workflow, which resulted in a final dataset of 92 cells after discarding units that did not meet acceptance criteria. Battery pack disassembly included removal of the enclosure, electrical disconnection, recovery of electronic components (e.g., sensors and control boards), inspection of mechanical condition, and isolation of individual cells as the minimum energy storage unit. Basic electrical measurements were recorded during screening to support dataset construction and subsequent analysis.

A key challenge in this process, and in second-life energy storage systems more broadly, is the heterogeneity of recovered cells. Cells may differ substantially in manufacturer, model, capacity, charge and discharge voltage profiles, weight, and chemical composition, which complicates the design and assembly of reliable second-life modules and packs.

The experimentation and testing process for 18650 cells typically begins with a review of manufacturer technical documentation, which reports nominal ratings and the corresponding test conditions. Because these conditions can differ substantially from real-world operating profiles, this step helps define a practical baseline for laboratory testing and subsequent data-driven analysis [25]. For example, the Panasonic NCR18650B datasheet specifies a standard charge current of 1.625 A to a terminal voltage of 4.2 V. After a full charge, the cell can be discharged at 0.65 A or 3.25 A to a cutoff voltage of 2.5 V, with reported discharge times of more than 300 min and 54 min, respectively.

Relevant international standards include IEC 62133, which specifies safety requirements and test methods for portable rechargeable cells and batteries [26]; IEC 62619, which addresses safety requirements for lithium-ion cells and batteries used in industrial applications, including tests under abusive conditions to reduce thermal, electrical, and mechanical risks [27]; and IEEE 1725, which provides design, validation, and safety requirements for lithium-ion battery systems used in portable devices [28].

The testing of 18650 cells recovered from laptop battery packs followed a sequential protocol designed to ensure safety, traceability, and reliable measurements. After extraction, each cell underwent visual inspection, was assigned a unique label, and was measured for open-circuit voltage to discard units showing swelling, corrosion, or voltages below 2.0 V. Cells that passed this initial screening were set to a storage voltage of 3.7 V and kept at room temperature (approximately 25 °C). Charge–discharge testing was then performed using constant-current/constant-voltage (CC–CV) cycles. Cells were charged at 0.5C or 1C to 4.20 V, followed by a constant-voltage stage until the current decreased to 0.05C. After a rest period to allow thermal stabilization, cells were discharged at 1C to a cutoff voltage of 3.0 V. During each cycle, discharged capacity, initial resting voltage, post-charge voltage, discharge time, and internal resistance were recorded. Internal resistance was estimated using a 10 s pulse at 1C (Quick Test). This protocol enabled SoH estimation, cell classification, and the construction of clustering models aimed at homogenizing key parameters for second-life repurposing.

In addition to the measured electrical variables, two health indicators were computed for each cell. The capacity-based state of health was defined as the ratio between the discharged capacity measured during testing and the nominal capacity reported for the corresponding commercial model:


$$\begin{array}{c} {SoH}_{i}=\frac{Q_{d,i}}{Q_{n,i}} \end{array}$$ (9)

Where *Q*_*d,i*_ (Ah) is the discharged capacity obtained during the test and *Q*_*n,i*_ (Ah) is the nominal rated capacity from the manufacturer datasheet. Dynamic internal resistance was estimated through the Quick Test procedure based on the voltage response to a controlled current pulse (*R* = *ΔV/ΔI*). For each cell, the resistance measurement was repeated three times and the average value was retained:


$$\begin{array}{c} R_{i}=\frac{\text{1}}{\text{3}}\sum_{j=\text{1}}^{\text{3}}R_{i,j} \end{array}$$ (10)

With *R*_*i*_ reported in milliohms (mΩ). To incorporate resistance-related degradation into the health assessment, an adjusted state of health metric was derived analytically from internal resistance by defining a resistance-based health term:


$$\begin{array}{c} \overset{\left(R\right)}{\underset{i}{SoH}}=\frac{R_{n,i}}{R_{i}} \end{array}$$ (11)

Where *R*_*n,i*_ is the model-level nominal internal resistance taken from the manufacturer datasheet for the corresponding commercial cell model and assigned to each recovered cell based on its identified model, and *R*_*i*_ is the measured average internal resistance. The adjusted state of health was then computed as a convex combination of the capacity-based and resistance-based terms:


$$\begin{array}{c} \overset{adj}{\underset{i}{SoH}}=w{SoH}_{i}+(\text{1}-w)\overset{\left(R\right)}{\underset{i}{SoH}},\;w=\text{0}.\text{6}\text{0}\text{3} \end{array}$$ (12)

The weighting coefficient w in the adjusted state of health (Eq. 12) balances the relative contribution of capacity-based SoH and resistance-based *SoH*_*R*_. A sensitivity analysis over w ∈ [0, 1] (101 equally spaced values) shows that the set of features passing the |r| ≥ 0.3 correlation threshold is stable for w ∈ [0.06, 0.84] (79 of 101 values), yielding {discharged capacity, discharge time, post-discharge voltage, average resistance}. After removing redundant pairs (|r| > 0.5), the two non-redundant clustering inputs — discharged capacity and average resistance — are retained for all w in this range. Because w = 0.603 lies well within the invariance interval, the feature selection and all downstream clustering results are insensitive to the specific value of w. This choice of capacity- and impedance-based indicators is consistent with our previous experimental analyses of 18650 cell degradation and performance using electrical measurements as health-related descriptors [29,30] Complete sensitivity curves are provided in S2 Fig and S2 Table.

**Fig 3:**
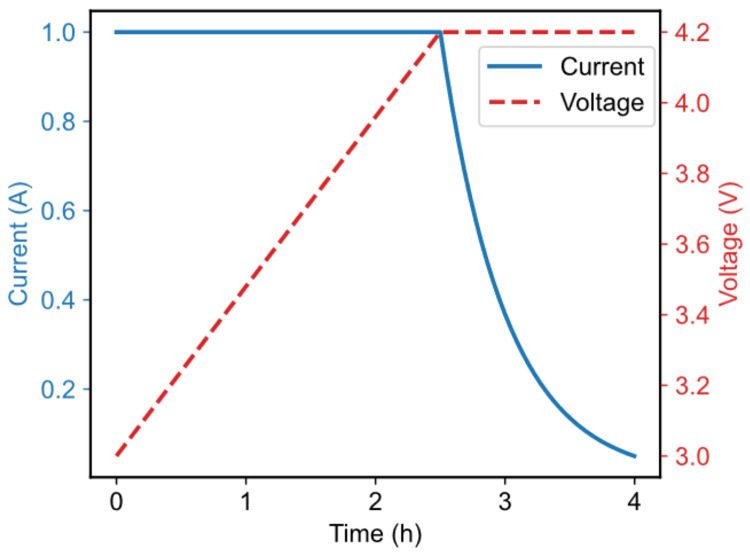
Representative CC/CV charging profile (current and voltage versus time) for an 18650 cell measured using the OPUS BT-C3400.

Electrochemical testing was performed using the charge, discharge, and quick-test modes of the OPUS BT-C3400 (manufacturer manual). In charge mode, cells were charged using a CC/CV algorithm at 1,000 mA until the terminal voltage reached 4.20 V. The device then held the voltage constant while the current decreased to its termination threshold, completing a controlled full charge. Fig 3 illustrates representative CC/CV waveforms. When high internal impedance was detected during charging, the device automatically reduced the current, enabling a more gradual charge for degraded cells.

In discharge mode, each cell was discharged to a predefined cutoff voltage (3.0 V) while the device displayed real-time current and recorded delivered capacity in milliampere-hours (mAh). The discharge current was set to 1,000 mA. This procedure provided an estimate of usable capacity under load, which was used for subsequent classification and selection for second-life applications. The ± 20% figure quoted in the OPUS BT-C3400 user manual is an upper bound under adverse contact conditions. To characterize the practical repeatability, we computed the coefficient of variation (CV) across the three replicate measurements for each of the 92 cells (Eq. 10). The median CV is 4.2% (mean = 5.8%, P95 = 13.4%); 63% of cells exhibit CV < 5% and only 3 cells (3.3%) exceed 20%. Averaging the three measurements further reduces the effective uncertainty by a factor of √3 ≈ 1.73. To assess propagation of this noise to pack-level outcomes, we performed a Monte Carlo experiment (B = 500) on the 91 cells retained for clustering, under two scenarios: (i) bootstrap of the three replicates per cell (reflecting actual protocol variability) and (ii) worst-case Gaussian perturbation with σ = 10% relative (interpreting ±20% as a 2-σ envelope). Under either scenario, the CV of aggregate pack capacity remains below 4.7% and the CV of mean resistance below 5.9%, with a Jaccard index of 0.62 (bootstrap) indicating that the majority of selected cells are stable across iterations. Because V1 and V2 are applied to the same measured dataset, this uncertainty affects both algorithms equally and the relative differences reported between them remain robust to resistance measurement error. Complete results are provided in S3 Fig and S3 Table.

The Quick Test mode was used to estimate the dynamic internal resistance of an 18650 cell by applying a controlled current pulse and measuring the resulting voltage response. This test takes approximately 10 s and reports resistance in milliohms (mΩ). To reduce state-of-charge effects, the test was performed only on fully charged cells.

**Fig 4:**
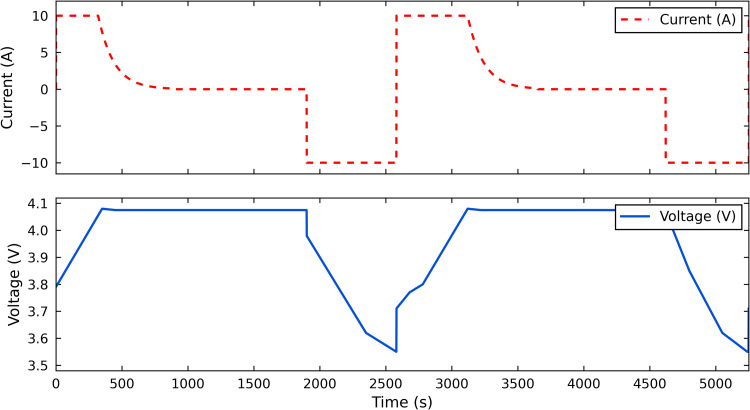
Current pulse profile and corresponding voltage response recorded during the quick test, used to estimate dynamic internal resistance of an 18650 cell.

Contact resistance at the terminals, leads, and charger slots can introduce measurement error; therefore, resistance estimates may vary by up to ~20% depending on the channel and contact conditions. Despite this variability, the Quick Test provides a rapid screening indicator of a cell’s ability to deliver current without excessive voltage drop, which is relevant for applications with transient power demands. In the evaluation of recovered cells, this measurement was used to identify units with unusually high internal resistance that could compromise the stability of a second-life battery pack. Fig 4 shows a representative current pulse profile and voltage response used to estimate internal resistance.

After screening and discarding unsuitable units (Fig 2), a final set of 92 cylindrical 18650 cells extracted from laptop battery packs was retained for analysis. For each cell, five parameters were recorded: initial resting voltage, voltage after a full CC/CV charge, discharged capacity, discharge time, and post-discharge voltage (recorded at the end of the discharge test; cutoff set to 3.0 V). The dataset includes cells from 16 commercial models manufactured by widely used brands, including Panasonic, Samsung, Sanyo, LG, and Sony. The identified chemistries span four main types: NCA (nickel cobalt aluminum), LCO (lithium cobalt oxide), NMC (nickel manganese cobalt), and LMO (lithium manganese oxide), capturing the diversity typically found in the portable electronics market.

**Fig 5:**
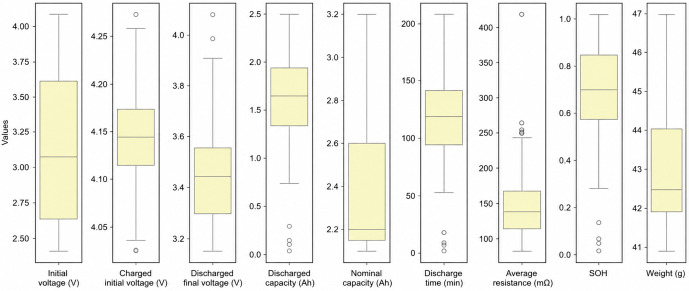
Box plots of the measured variables for the 92 retained 18650 cells.

The mean initial voltage was 3.15 V (SD = 0.55 V), while the fully charged voltage was 4.145 V (SD = 0.053 V). Discharged capacity averaged 1.618 Ah (SD = 0.533 Ah), with a mean discharge time of 116.6 min (SD = 40 min). The post-discharge voltage averaged 3.456 V (SD = 0.197 V), where post-discharge voltage refers to the voltage recorded at the end of the discharge test for each cell (the discharge cutoff was set to 3.0 V). These data provide a quantitative characterization of cell-to-cell variability and support subsequent clustering analysis and modeling of health-related indicators. Fig 5 summarizes the distribution of the measured variables using box plots. The results show marked heterogeneity; for example, discharged capacity averaged 1.62 Ah (SD = 0.53 Ah) and ranged from 0.038 Ah to 2.50 Ah (range = 2.46 Ah).

Fig 5 summarizes the distributions of the measured variables for the 92 retained cells and highlights substantial heterogeneity. For example, discharged capacity averaged 1.62 Ah (SD = 0.53 Ah) and ranged from 0.038 Ah to 2.50 Ah (range = 2.46 Ah).

This variability is also evident in discharge time, which ranges from 2 to 208 min and has a standard deviation of more than 40 min, indicating substantial differences in cell performance under the same test conditions. Internal resistance, measured three times per cell, also showed marked dispersion. Mean resistance values were approximately 145–150 mΩ, with standard deviations close to 50 mΩ and ranges as large as 374 mΩ, reflecting large differences in internal impedance and, consequently, in expected electrical and thermal losses. The average of the three resistance measurements reached a maximum of 418.33 mΩ, more than three times the minimum recorded value.

Nominal capacity showed less dispersion (SD = 0.33 Ah) but still reflected multiple cell designs, ranging from 2.1 Ah to 3.2 Ah. SoH also varied widely, with a mean of 0.68 (SD = 0.22) and a range of 1.0, spanning from severely degraded cells (SoH ≈ 0.02) to cells with capacity close to their nominal rating (SoH ≈ 1.0).

Table 4 summarizes descriptive statistics for the 92 retained 18650 cells used to construct the dataset for second-life applications. The dispersion across key variables motivates the use of methods that support homogeneous segmentation and grouping of recovered cells. Clustering algorithms are suitable for this purpose because they organize cells into groups with similar electrical and functional characteristics, which supports the design of more balanced second-life battery packs and more consistent pack-level behavior.

**Table 4:** Descriptive statistics of the measured variables for the 92 retained 18650 cells.

	Mean	SD	Min	Q1	Median	Q3	Max	Variance	Range	CV (SD/Mean)
**Initial Voltage (V)**	3.149	0.546	2.410	2.635	3.075	3.613	4.085	0.299	1.675	0.174
**Fully Charged Voltage (V)**	4.145	0.053	4.025	4.115	4.144	4.174	4.273	0.003	0.248	0.013
**Post-Discharge Voltage (V)**	3.455	0.193	3.152	3.298	3.444	3.555	3.984	0.037	0.832	0.056
**Discharged Capacity (Ah)**	1.618	0.533	0.038	1.337	1.648	1.935	2.496	0.284	2.458	0.329
**Nominal Capacity (Ah)**	2.401	0.328	2.100	2.150	2.200	2.600	3.200	0.107	1.100	0.136
**Discharge Time (min)**	116.598	40.061	2.000	94.500	119.000	141.250	208.000	1604.880	206.000	0.344
**Average Resistance (mΩ)**	147.688	51.404	83.000	114.667	138.333	167.667	418.333	2642.337	335.333	0.348
**State of Health (SoH)**	0.676	0.216	0.017	0.575	0.700	0.845	1.018	0.047	1.001	0.320
**Weight (g)**	43.019	1.585	40.900	41.921	42.472	44.031	46.964	2.512	6.064	0.037

**Fig 6:**
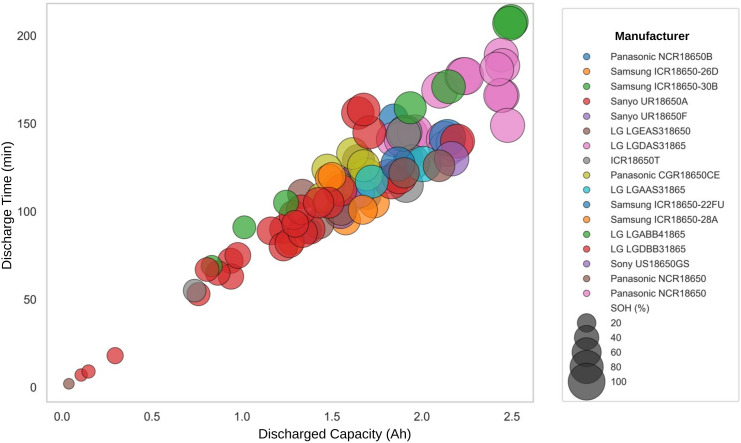
Discharged capacity versus discharge time for the 92 retained 18650 cells, with bubble size proportional to SoH (%) and color indicating manufacturer and model.

Fig 6 summarizes the performance of the cells in the dataset using a bubble chart of discharged capacity (Ah) versus discharge time (min). Bubble size is proportional to State of Health (SoH, %), and colors distinguish cell manufacturer and model. The plot highlights the heterogeneity of the recovered cells and provides a visual basis for identifying groups with similar behavior.

## Results

This section reports the outcomes of the automated workflow used to select 18650 lithium-ion cells recovered from laptop batteries and to support the assembly of second-life battery modules or packs. The aim was to obtain a set of cells that is as homogeneous as possible with respect to critical characteristics (e.g., discharged capacity and state of health), supporting consistent performance during pack construction. The results are presented for the correlation analysis, the comparison of clustering algorithms, the initial clustering stage, and the iterative selection step.

### Correlation analysis

**Fig 7:**
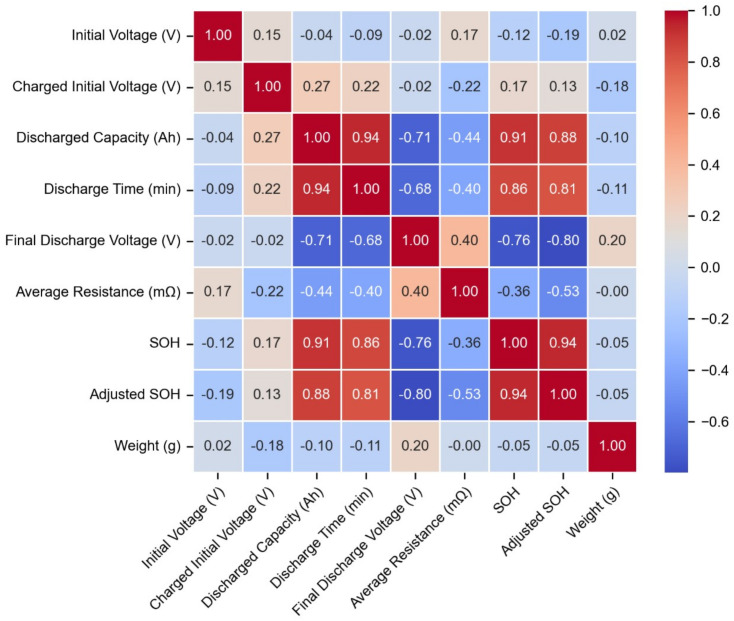
Pearson correlation heatmap for the measured variables and health metrics (SoH and Adjusted SoH) in the 92 retained 18650 cells.

As a first step, Pearson correlations were examined to identify variables most strongly associated with cell health metrics (SoH and Adjusted SoH) and to reduce dimensionality before clustering. Fig 7 summarizes the correlation structure of the dataset.

Discharged capacity and discharge time are strongly and positively correlated with SoH (r = 0.91 and r = 0.86, respectively) and with Adjusted SoH (r = 0.88 and r = 0.81). post-discharge voltage is strongly and negatively correlated with both SoH and Adjusted SoH (r = −0.76 and r = −0.80). Average resistance shows moderate negative correlations with SoH (r = −0.36) and Adjusted SoH (r = −0.53). By contrast, initial voltage, charged voltage, and weight show weak correlations with the health metrics (|r| ≤ 0.19). This inverse relationship is expected because low-SoH cells typically reach the cutoff condition earlier and deliver less capacity; when the load is removed, their voltage rebounds and remains relatively high due to the higher remaining state of charge.

Given the redundancy between discharged capacity and discharge time (r = 0.94), the clustering stage retained discharged capacity as the primary energy-delivery descriptor and average resistance as a complementary degradation-related descriptor. This reduced, non-redundant feature set supports the formation of more homogeneous groups for second-life pack construction.

### Clustering algorithm performance

This section compares K-means with a Gaussian Mixture Model (GMM) initialized with K-means under two covariance structures (full and spherical). Performance was evaluated using the average Silhouette index for k values from 2 to 10 on the retained dataset (n = 92).

#### Initial clustering.

**Fig 8:**
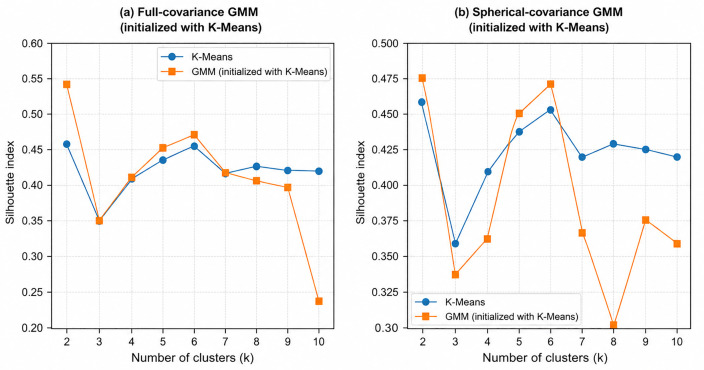
Average Silhouette index versus number of clusters (k) for K-means and GMM initialized with K-means (n = 91): (a) full-covariance GMM; (b) spherical-covariance GMM.

Fig 8 compares the average Silhouette index across k = 2–10 for K-means and GMM initialized with K-means under two covariance structures. Under full covariance (Fig 8a), both methods follow similar trends, with GMM performing slightly better for 2 ≤ k ≤ 6 and both reaching their peak at k = 6 (GMM: 0.466; K-means: 0.460); at k = 10, the full-covariance GMM exhibits a pronounced drop while K-means remains stable (0.42–0.47). Under spherical covariance (Fig 8b), GMM outperforms K-means at k = 2 (0.475 vs. 0.457) and at k = 6 (0.472 vs. 0.460), while K-means is superior at intermediate values (k = 3–5); as in the full-covariance case, the spherical GMM deteriorates at higher k. In both configurations, k = 6 yields the highest overall Silhouette score and was therefore selected as the initial partition size for the subsequent cell selection stage.

**Fig 9:**
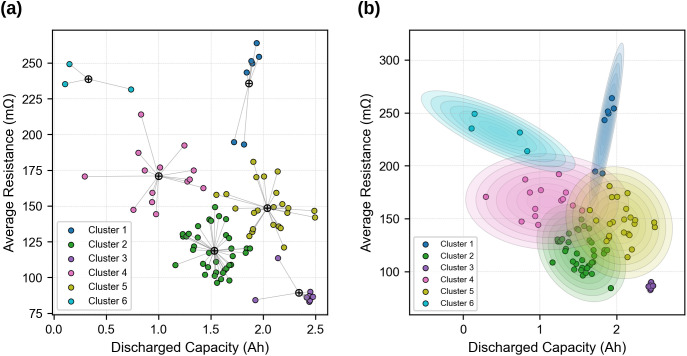
Clustering results at k = 6 in the discharged capacity–average resistance plane: (a) K-means partition; (b) GMM partition initialized with K-means, showing Gaussian component dispersion.

To visualize the partition at k = 6, Fig 9a shows the K-means solution in the feature space defined by discharged capacity (x-axis) and average resistance (y-axis), with points colored by cluster assignment. Fig 9b presents the corresponding GMM solution initialized from the same centroids, where Gaussian components provide an additional view of cluster dispersion relative to the centroid-based K-means partition.

#### Iterative subclustering.

Subclustering was conducted within the six groups produced by the initial full-covariance GMM partition (k = 6). Within each parent cluster, k was varied from 2 up to a maximum permitted by cluster size, and Silhouette scores were recomputed to assess potential gains in within-group homogeneity.

Across very small clusters (e.g., clusters 1, 3, and 6), K-means and GMM produced identical Silhouette scores for all tested k values, indicating limited benefit from additional probabilistic modeling when sample size is small. In contrast, improvements emerged for medium-sized clusters. For cluster 2 (37 cells), the full-covariance GMM slightly outperformed K-means at k = 2 (0.451 vs. 0.439), while the spherical GMM reached a higher peak at k = 5 (0.478), exceeding the corresponding K-means score (0.402). The largest gains were observed in cluster 5 (23 cells), where full-covariance GMM achieved 0.510 at k = 6 and remained higher than K-means across k = 5–9, and spherical GMM reached the highest subclustering Silhouette score (0.522 at k = 8).

Overall, these results indicate that GMM, particularly under the spherical covariance assumption, can improve subclustering quality in clusters of intermediate size, while offering negligible advantages in very small groups. This supports the use of a combined K-means and GMM strategy to refine homogeneity prior to second-life pack assembly. Post-hoc analysis of the correspondence between cluster membership and cell chemistry (Table 5) reveals that the GMM partition does not cleanly separate chemical families: the Adjusted Rand Index is 0.158 and the Normalized Mutual Information is 0.290. Two small clusters are chemistry-pure (cluster 1: 6 NCA cells; cluster 5: 5 LCO cells), while the working cluster (cluster 2, 34 cells) contains all four chemistries, achieving only 44% purity. The weighted global purity is 0.659. This outcome is expected and, in the context of second-life reuse, desirable: because the clustering variables are discharged capacity and average resistance — both reflecting the current electrochemical state rather than the original chemistry — the partition groups cells by actual performance, not by manufacturer label. Cells of the same chemistry end up in different clusters when their aging histories diverge, which is precisely the heterogeneity that the selection algorithms must navigate.

**Table 5:** Cross-tabulation of GMM cluster membership and cell chemistry for the 91 retained cells.

Cluster	LCO	NMC	NCA	LMO	Total	Dominant	Purity (%)
0	6	7	0	0	13	NMC	54
1	0	0	6	0	6	NCA	100
2	9	15	4	6	34	NMC	44
3	2	2	0	0	4	Mixed	50
4	25	1	2	1	29	LCO	86
5	5	0	0	0	5	LCO	100
Total	47	25	12	7	91	–	65,9

Cross-tabulation of GMM cluster membership (k = 6) and cell chemistry for the 91 retained cells. Purity is defined as the proportion of the dominant chemistry within each cluster. ARI = 0.158; NMI = 0.290; weighted global purity = 0.659.

### Battery packs obtained through the selection algorithms

Based on the iterative clustering workflow, second-life battery packs were assembled by selecting subsets of cells with high within-group homogeneity. Packs produced by each selection algorithm were then compared.

#### Battery packs from selection algorithm version 1.

Selection Algorithm Version 1 implements an iterative, Silhouette-driven strategy that selects cells from the most cohesive partition compatible with the target pack topology. The method evaluates candidate partitions by varying the number of clusters *k* from the minimum required by the topology (*k = p*, where *p* is the number of parallel groups) up to a maximum value constrained by the available data. For each *k*, a Gaussian mixture model (GMM) initialized with K-means is fitted to obtain a partition.

**Fig 10:**
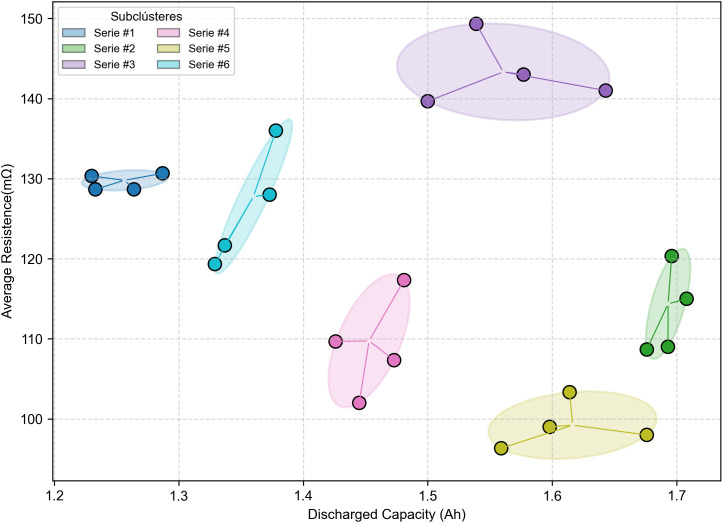
Selected groups produced by selection algorithm version 1 in the space of discharged capacity (Ah) and average internal resistance (mΩ).

Partition quality is assessed using the global Silhouette index together with per-cell Silhouette scores. Clusters that contain at least the required number of cells are retained; the *p* most cohesive clusters are then selected based on their mean Silhouette score. Within each selected cluster, cells are ranked by their individual Silhouette scores, and the top *s* cells are chosen to form each series string (where *s* is the number of cells in series per parallel group). Fig 10 shows the resulting six groups for the case study cluster used in this stage, displayed in the feature space of discharged capacity (x-axis) and average internal resistance (y-axis).

**Fig 11:**
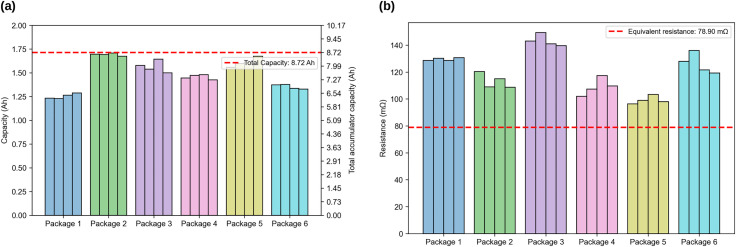
Pack-level consistency for selection algorithm version 1: (a) Cell capacities by package and total pack capacity; (b) Cell internal resistances by package and equivalent pack resistance.

**Fig 12:**
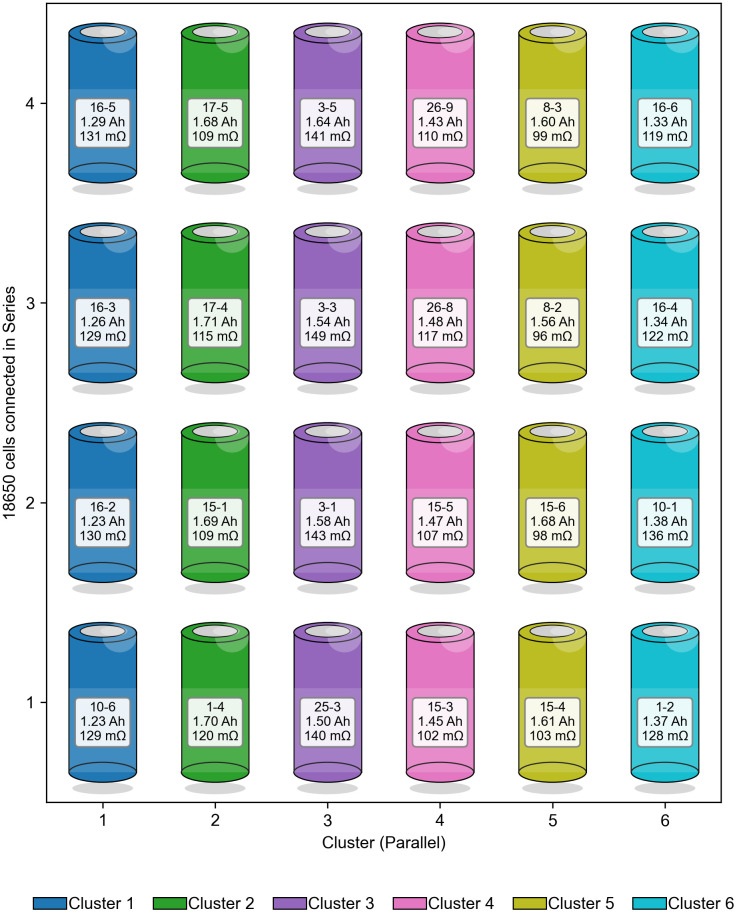
Final pack configuration obtained with selection algorithm version 1, showing the selected cell identifiers and their measured capacity and resistance.

Fig 11 summarizes the electrical consistency of the assembled pack. Fig 12a reports the capacity of the selected cells grouped by package (left axis) and the resulting total pack capacity (right axis). Fig 12b reports the internal resistance of the selected cells by package and the equivalent internal resistance of the assembled pack.

Finally, Fig 12 presents the final pack layout with each selected cell identified by its original label, confirming traceability from the experimental dataset to the assembled configuration.

#### Battery packs from selection algorithm version 2.

Selection Algorithm Version 2 follows the same GMM-based selection framework as Version 1 but adds a hierarchical refinement step to identify more cohesive subsets of cells. Instead of relying on a single global partition, this strategy progressively subdivides eligible clusters to explore partitions with higher within-group homogeneity.

After an initial partition is obtained, each cluster is evaluated based on its size to determine whether it can be subdivided into valid subgroups that satisfy the minimum cardinality required by the target pack topology. The purpose of this step is to expand the pool of candidate groups by revealing latent structure that may be masked in the first partition. Subgroups that meet the size constraint and improve cohesion are retained as candidates for pack assembly. From this candidate pool, the most cohesive non-overlapping groups are selected, and within each group the most representative cells are chosen to form the series-connected strings of the final pack.

**Fig 13:**
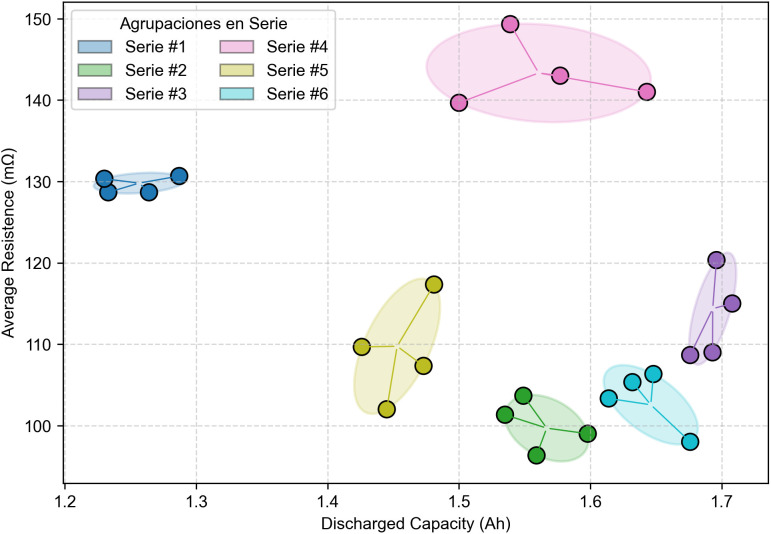
Cell groups selected by selection algorithm version 2 in the discharged capacity–average resistance space (applied to cluster 2 from the initial clustering stage).

Fig 13 shows the six selected series groups obtained with Version 2 (illustrated here for Cluster 2 from the clustering performance analysis), using discharged capacity and average resistance as the two-dimensional selection space.

**Fig 14:**
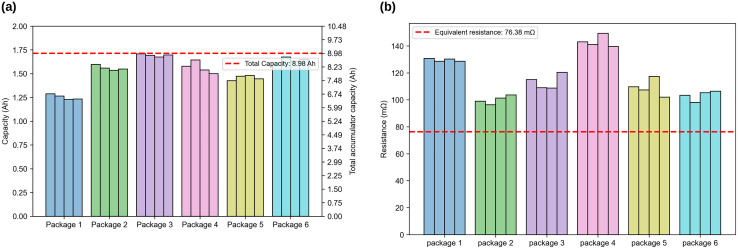
Pack characteristics obtained with selection algorithm version 2: (a) Cell capacities and total pack capacity; (b) Cell internal resistances and equivalent pack resistance.

Fig 14 summarizes the resulting pack characteristics. Fig 14a reports the capacity of the selected cells and the total pack capacity, while Fig 14b reports cell-level internal resistance and the equivalent pack resistance.

**Fig 15:**
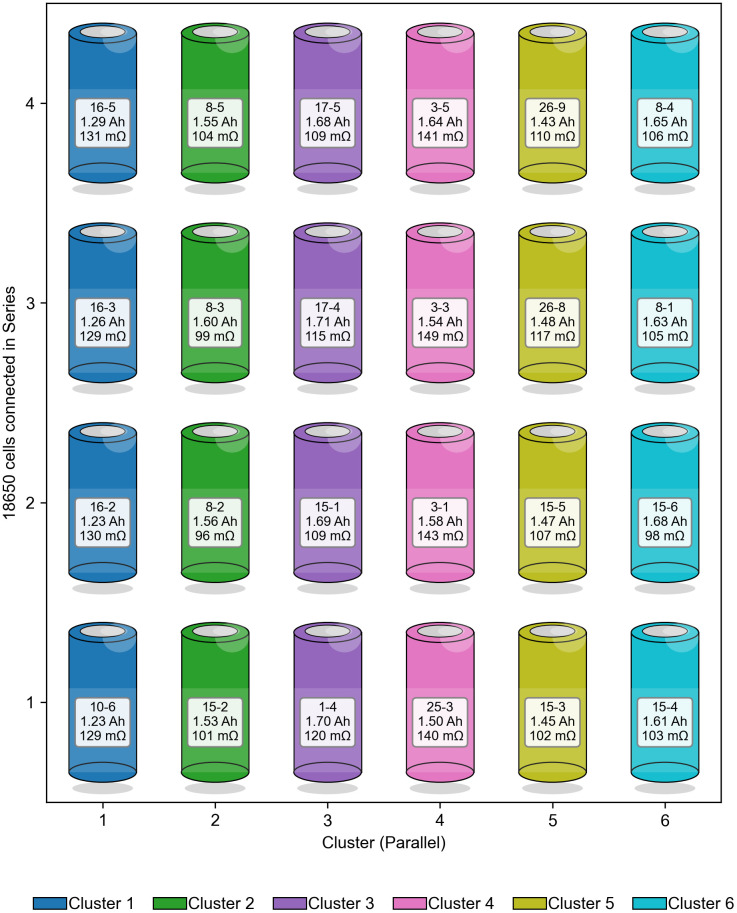
Final second-life pack assembled using selection algorithm version 2, showing cell identifiers for traceability.

Fig 15 provides the final pack layout with cell identifiers, enabling traceability between the selected cells and their original labels. This graphical representation allows for validation that the cells selected through the hierarchical refinement process remain consistent with their original labeling, ensuring traceability within the final battery pack.

The estimated maximum power was computed under the maximum power transfer theorem as $Pmax\;=\;Vpack^{2}\;/\;(\text{4}\;\cdot \;Req)$, where Vpack = s · Vnom (with Vnom = 3.7 V per cell) and *R*_*eq*_ is the equivalent pack resistance computed from the series–parallel combination of individual cell resistances. This estimate assumes a matched load and does not account for thermal limits, connector losses, or voltage cutoffs; it serves as a comparative metric between V1 and V2 rather than an operational specification.

#### Statistical comparison with random-selection baseline.

To assess whether the clustering-guided selection provides a statistically meaningful advantage, we generated a random-selection baseline by constructing N = 10,000 packs from the working cluster (37 cells), each time drawing 24 cells at random and assigning them to six strings of four. Table 6 reports the resulting distribution alongside the V1 and V2 outcomes. Both V1 and V2 exceed the 95th percentile of the random baseline in pack capacity (V1: 99.8th percentile, 8.72 Ah; V2: 100th percentile, 8.98 Ah), confirming that the clustering-based selection yields packs that are statistically superior to random assembly. The improvement is particularly pronounced for V2 in resistance and maximum power, where it reaches the 93rd percentile of the random distribution (76.38 mΩ and 716.97 W, respectively), compared with V1 which ranks near the median for these metrics. The effect size relative to the random baseline is large to very large (Cohen’s d = 2.96–4.12 for capacity; d = 1.51 for V2 resistance). The V2–V1 difference, taken in isolation, does not reach conventional statistical significance (capacity: p = 0.42; resistance: p = 0.15; power: p = 0.15), which is expected given that both algorithms select 24 out of 37 available cells (65%), leaving limited room for differentiation. The consistent directional advantage of V2 across all three metrics, combined with its substantially higher ranking in the random-baseline distribution, supports the conclusion that hierarchical refinement improves pack assembly quality, particularly in resistance homogeneity. Complete numerical results for this analysis are provided in S4 Table.

**Table 6:** Pack-level metrics for Selection Algorithms V1 and V2 compared against a random-selection baseline (N = 10,000).

Metric	Random baseline (mean ± SD)	95% CI	V1	V1 percentile	V2	V2 percentile
Pack capacity (Ah)	8.05 ± 0.22	[7.65, 8.52]	8.72	99.8th	8.98	100th
Equiv. resistance (mΩ)	78.26 ± 1.25	[75.84, 80.70]	78.90	31st	76.38	93rd
Max. power (W)	699.9 ± 11.2	[678.5, 722.0]	694.08	31st	716.9	93rd
Cohen’s d (capacity)	—	—	2.96	—	4.12	—
Cohen’s d (resistance)	—	—	−0.51	—	1.51	—

Comparison of V1 and V2 pack-level metrics against a random-selection baseline (N = 10,000 packs assembled from 37 cells in the working cluster, 4s6p topology). Percentiles indicate the position of each algorithm’s outcome within the random distribution. Cohen’s d is computed relative to the random baseline mean and standard deviation.

Table 7 compares the main pack-level metrics obtained with Versions 1 and 2. Version 2 increases total system capacity from 8.72 Ah to 8.98 Ah (+2.98%) and reduces equivalent internal resistance from 78.90 mΩ to 76.38 mΩ (−3.19%). Consistent with these changes, the estimated maximum power increases from 694.08 W to 716.97 W (+3.30%). Overall, the hierarchical refinement in Version 2 yields a slightly more homogeneous and electrically efficient cell set, which translates into improved pack-level consistency and performance.

**Table 7:** Pack-level electrical metrics for the battery packs assembled using selection algorithm versions 1 and 2.

Property	Version 1	Version 2	Variation (%)
Total system capacity (Ah)	8.72	8.98	+2.98
Internal resistance (mΩ)	78.90	76.38	–3.19
Maximum power (W)	694.08	716.97	+3.30

## Discussion

This work addressed a practical barrier in second-life battery reuse: recovering cells from heterogeneous first-life sources and assembling a pack with sufficiently consistent behavior to support reliable operation. The experimental dataset confirmed substantial variability across recovered 18650 cells in both energy-delivery behavior (discharged capacity and discharge time) and degradation-related behavior (internal resistance and SoH). This heterogeneity motivates an algorithmic selection stage prior to assembly, because cell-to-cell imbalance can translate into uneven loading, accelerated aging, and reduced usable capacity at the pack level.

Correlation analysis supported the use of a reduced, non-redundant feature set for clustering. Discharged capacity and discharge time were strongly correlated with SoH and with each other, indicating redundancy between these two descriptors under the adopted test protocol. Retaining discharged capacity as the primary energy-related variable while incorporating average internal resistance as a complementary degradation-related variable provides an interpretable basis for regrouping: capacity captures usable charge delivery, whereas resistance reflects impedance growth and expected losses. This pairing is also aligned with second-life practice, where capacity screening alone may overlook cells that appear acceptable by capacity but exhibit elevated resistance that limits power capability and increases heat generation.

Across the tested values of *k*, the comparison between K-means and GMM, with GMM initialized from the K-means solution, indicates that probabilistic modeling can improve partition quality in certain settings, particularly for groups with intermediate variability that are large enough to benefit from the added model flexibility. In very small groups, the advantage of GMM is limited, as parameter estimation becomes less stable and the clustering structure can be influenced by a small number of points. From a practical standpoint, these findings suggest that a hybrid workflow that uses K-means for initialization and GMM for refinement can be useful when recovered-cell datasets depart from a purely centroid-separable structure because they combine multiple cell models and heterogeneous aging histories.

A key contribution of this study is moving beyond regrouping as an abstract outcome and evaluating the consequences at the pack level through physical assembly and testing. The two selection algorithms produced packs with measurable differences in aggregate electrical properties. Version 2, which introduces hierarchical refinement, yielded a consistent improvement relative to Version 1: higher total pack capacity (+2.98%), lower equivalent internal resistance (–3.19%), and higher estimated maximum power (+3.30%). While these deltas are modest, they are meaningful in the second-life context, where gains in usable capacity and reductions in resistance translate into improved efficiency, lower voltage sag under load, and potentially reduced thermal stress. Importantly, the improvement was achieved without increasing the number of cells or changing the pack topology, indicating that refinement contributes value primarily through improved homogeneity of the selected cell set. The random-baseline comparison (Table 5) demonstrates that the primary contribution of the proposed workflow is the clustering-guided selection itself, rather than the incremental refinement between V1 and V2. Both algorithms produce packs that are statistically distinguishable from random assembly (both outside the 95% confidence interval for capacity), with effect sizes in the large-to-very-large range (Cohen’s d > 2.9). The V2 refinement adds practical value by shifting the pack from the median to the 93rd percentile in resistance — a metric directly linked to thermal uniformity and degradation rate in parallel-connected cells [31]. While the V2–V1 difference does not independently reach p < 0.05, this is a consequence of the constrained selection ratio (24/37 = 65%) rather than a lack of meaningful improvement; in larger recovered-cell inventories the selection ratio would decrease and the statistical power of the comparison would increase accordingly.

Several measurement aspects should be considered when interpreting the results. Internal resistance was estimated using a short pulse method, which is sensitive to contact resistance and state-of-charge effects. Although averaging repeated measurements reduces random error, absolute resistance values may still carry systematic uncertainty. In addition, nominal specifications (capacity and nominal resistance) were taken from manufacturer datasheets and assigned based on identified commercial models; this is a pragmatic approach for mixed-source recovery, but it introduces uncertainty when model identification is ambiguous or when datasheet conditions differ from the adopted test protocol. The adjusted SoH metric partially mitigates this by combining capacity and resistance information, but its behavior depends on the reliability of these inputs and the weighting strategy. Overall, the present study provides a practical and reproducible workflow that links laboratory screening, clustering-guided selection, and pack assembly to improve homogeneity in second-life battery construction. A more comprehensive characterization based on electrochemical impedance spectroscopy (EIS) would decompose the internal resistance into ohmic, charge-transfer, and diffusion components, providing additional insight into specific aging modes. We did not perform EIS in this work because (i) the clustering step relies on the relative ranking of cells under min–max normalization rather than absolute resistance values, and Quick Test and EIS-derived total resistance are typically rank-correlated for cells of similar form factor and chemistry; and (ii) EIS measurement times of tens of minutes per cell and the associated instrumentation cost are not aligned with the scalable second-life screening context targeted by this study. A direct comparative analysis between Quick Test and DCIR/EIS on a subset of cells is identified as a natural extension of this work.

The computational cost of the proposed workflow scales as O(n·k·i) for K-means and GMM fitting (where n is the number of cells, k the number of clusters, and i the number of EM iterations) plus O(n^2^) for Silhouette evaluation. For the 91-cell dataset used in this study, the complete V2 pipeline executes in under 2 seconds on a standard laptop (Intel i7, 16 GB RAM). Extrapolation to industrial inventories of 1,000–10,000 cells is feasible without algorithmic modification: at n = 10,000 the Silhouette computation dominates at approximately 10–15 seconds, while the clustering step remains sub-second. For inventories exceeding 50,000 cells, approximate Silhouette methods (mini-batch sampling) or alternative validation indices with O(n·k) complexity could replace the exact computation without affecting the selection logic.

Scope and limitations. This study provides a static characterization and single-point comparison of pack-level metrics. While the random-baseline analysis demonstrates that clustering-guided selection produces statistically superior packs, the present work does not include cycling tests, thermal monitoring, or accelerated aging experiments. The reported improvements in capacity (+2.98%) and resistance (−3.19%) reflect initial assembly quality, not guaranteed long-term performance. Theoretical and empirical evidence from the literature supports the expectation that initial homogeneity reduces differential aging: Bruen and Marco [31] showed that a 30% impedance mismatch among parallel cells causes up to 60% current imbalance and 6% charge-throughput disparity, while Odabaşı et al. [32] demonstrated an 8% SoH advantage after 1,000 cycles for sorted versus unsorted modules. Extrapolating from these findings, the resistance-homogeneity improvement achieved by V2 (from the 31st to the 93rd percentile of the random baseline) is expected to yield a measurable reduction in degradation rate, although the magnitude requires empirical validation under representative load profiles. A direct cycling comparison of V1 and V2 packs, measurement improvements such as standardized resistance methods and impedance characterization, and assessment across larger datasets and additional form factors are identified as the primary extensions of this work.

## Conclusions

This study proposed and experimentally validated a practical workflow for second-life 18650 cell reuse that connects cell characterization, clustering-based regrouping, and physical pack assembly. Using recovered laptop cells, the analysis showed that discharged capacity and average internal resistance provide a compact, non-redundant feature set that captures both energy-delivery capability and degradation-related effects relevant to second-life selection. A comparative evaluation of K-means and GMM (initialized from K-means) indicated that probabilistic clustering can offer advantages in partitions with intermediate variability, supporting its use within an iterative selection framework.Two iterative selection strategies were implemented and compared through pack construction and testing. The hierarchical refinement strategy (Version 2) produced a pack with improved aggregate electrical performance relative to Version 1, including higher total capacity (+2.98%), lower equivalent internal resistance (–3.19%), and higher estimated maximum power (+3.30%). A random-selection baseline (N = 10,000) confirmed that both algorithms produce packs that are statistically superior to random assembly (V1: 99.8th percentile in capacity; V2: 100th), with large effect sizes (Cohen’s d = 2.96–4.12). The results demonstrate that clustering-guided cell selection produces second-life packs with statistically superior initial electrical characteristics compared to random assembly. The hierarchical refinement in V2 specifically improves resistance homogeneity, a factor linked to reduced differential aging in the literature. These findings should be interpreted as proof-of-concept evidence for a static assembly metric; validation under cyclic loading and thermal stress remains necessary before deployment recommendations can be made.

## Supporting information

S1 Table Seed-sensitivity analysis results. Silhouette index and pairwise Adjusted Rand Index across 100 random states for K-means at k = 6, comparing *n*_*init*_ = 1 and *n*_*init*_ = 50.
(XLSX)

S2 Table Sensitivity of feature selection to the weighting coefficient w. Pearson correlation between each candidate feature and the adjusted SoH for 101 values of w ∈ [0, 1].
(XLSX)

S3 Table OPUS BT-C3400 repeatability statistics. Coefficient of variation across three replicate resistance measurements for each of the 92 cells.
(XLSX)

S4 Table Random-selection baseline and statistical significance results. Distribution of pack metrics across N = 10,000 random packs, percentile ranking of V1 and V2, p-values, and Cohen’s d effect sizes.
(XLSX)

S5 Table Cross-tabulation of cluster membership by cell chemistry and manufacturer. Cell counts per cluster for each of the four chemistries (LCO, NMC, NCA, LMO) and 17 commercial models.
(XLSX)

S6 Table Hyperparameter specification for Selection Algorithms V1 and V2. Complete list of parameters, values, and justifications for all clustering and selection steps.
(XLSX)

S1 Appendix Detailed Gaussian mixture model formulation. Probability density function, covariance matrix structure, log-likelihood, and expectation–maximization parameter update equations (E-step and M-step).
(DOCX)

S1 Fig Distribution of Silhouette scores across 100 random seeds. Box plots comparing *n*_*init*_ = 1 and *n*_*init*_ = 50 initialization protocols.
(TIFF)

S2 Fig Feature correlation curves as a function of w. Visualization of the invariance interval [0.06, 0.84] within which the selected feature set remains unchanged.
(TIFF)

S3 Fig Monte Carlo propagation of resistance measurement noise to pack-level metrics. Distribution of pack capacity, resistance, and Jaccard index under bootstrap and worst-case scenarios (B = 500).
(TIFF)
