# Supplementary figures and images for: Clustering recovered 18650 lithium-ion cells to improve homogeneity in second-life battery pack construction
Source: PLoS One. 2026 Jul 16;21(7):e0353394. doi: 10.1371/journal.pone.0353394 (PMC13374983; doi:10.1371/journal.pone.0353394)

**S1 Appendix. Detailed GMM formulation.**


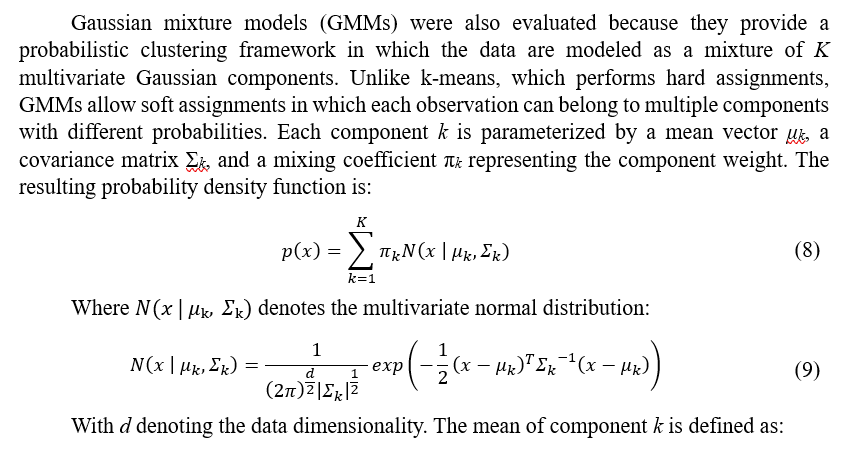


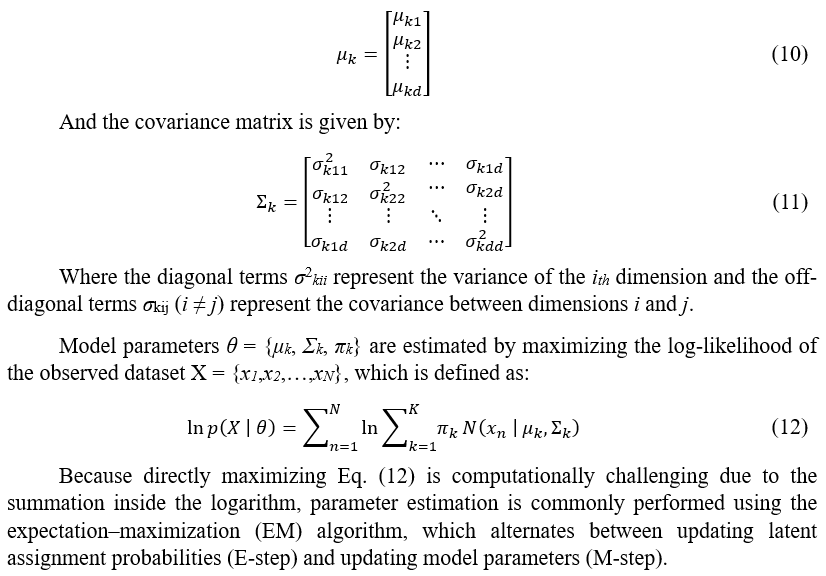


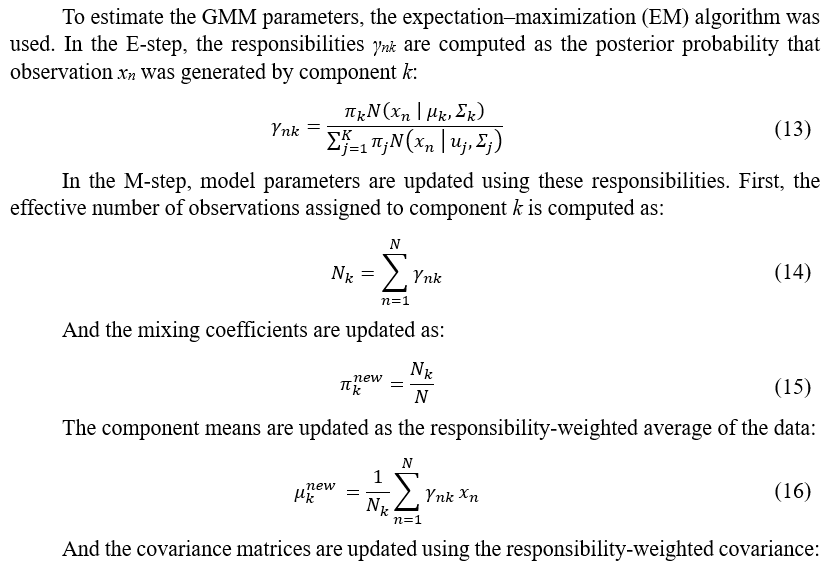


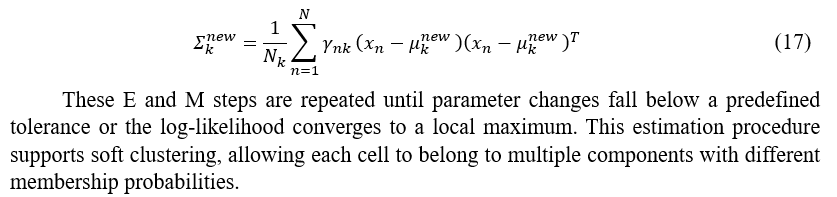

Supplement: S1 Appendix — Probability density function, covariance matrix structure, log-likelihood, and expectation–maximization parameter update equations (E-step and M-step). (DOCX) [file pone.0353394.s007.docx]

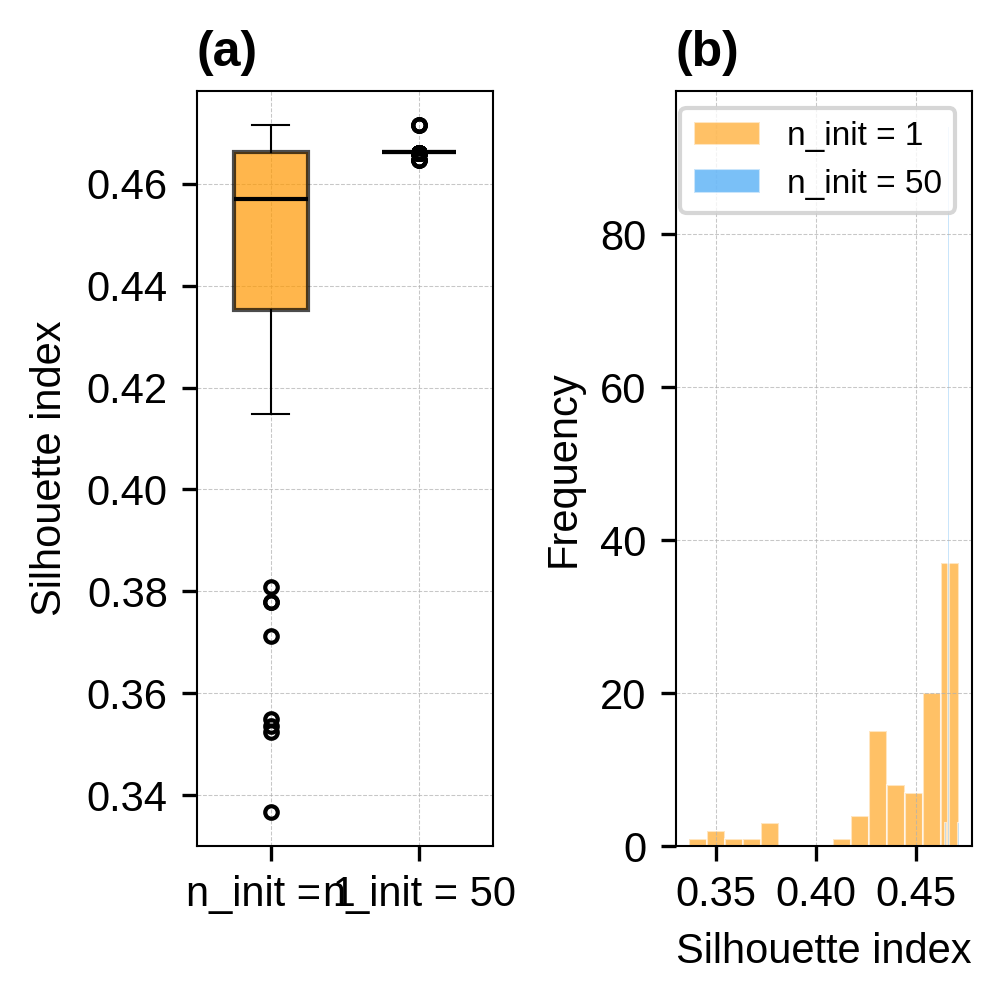

Supplement: S1 Fig — Box plots comparing ninit = 1 and ninit = 50 initialization protocols. (TIFF) [file pone.0353394.s008.tiff]

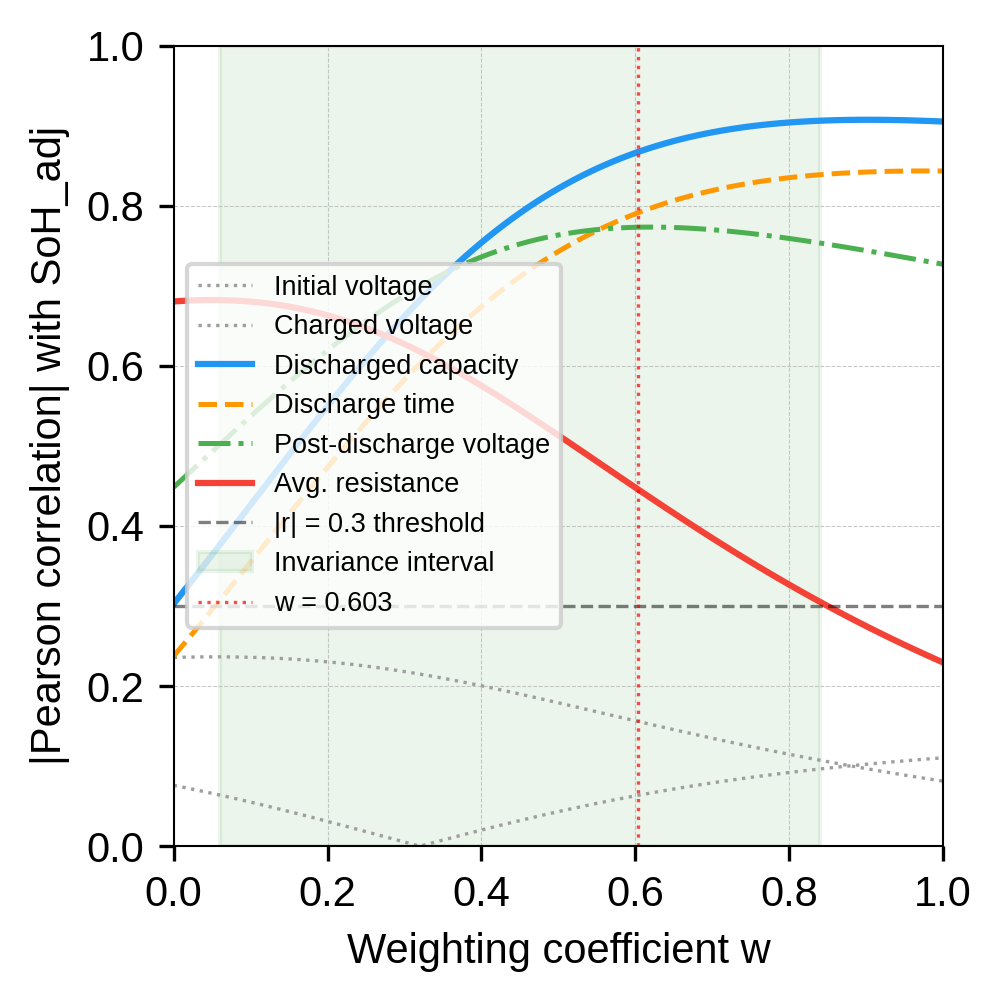

Supplement: S2 Fig — Visualization of the invariance interval [0.06, 0.84] within which the selected feature set remains unchanged. (TIFF) [file pone.0353394.s009.tiff]

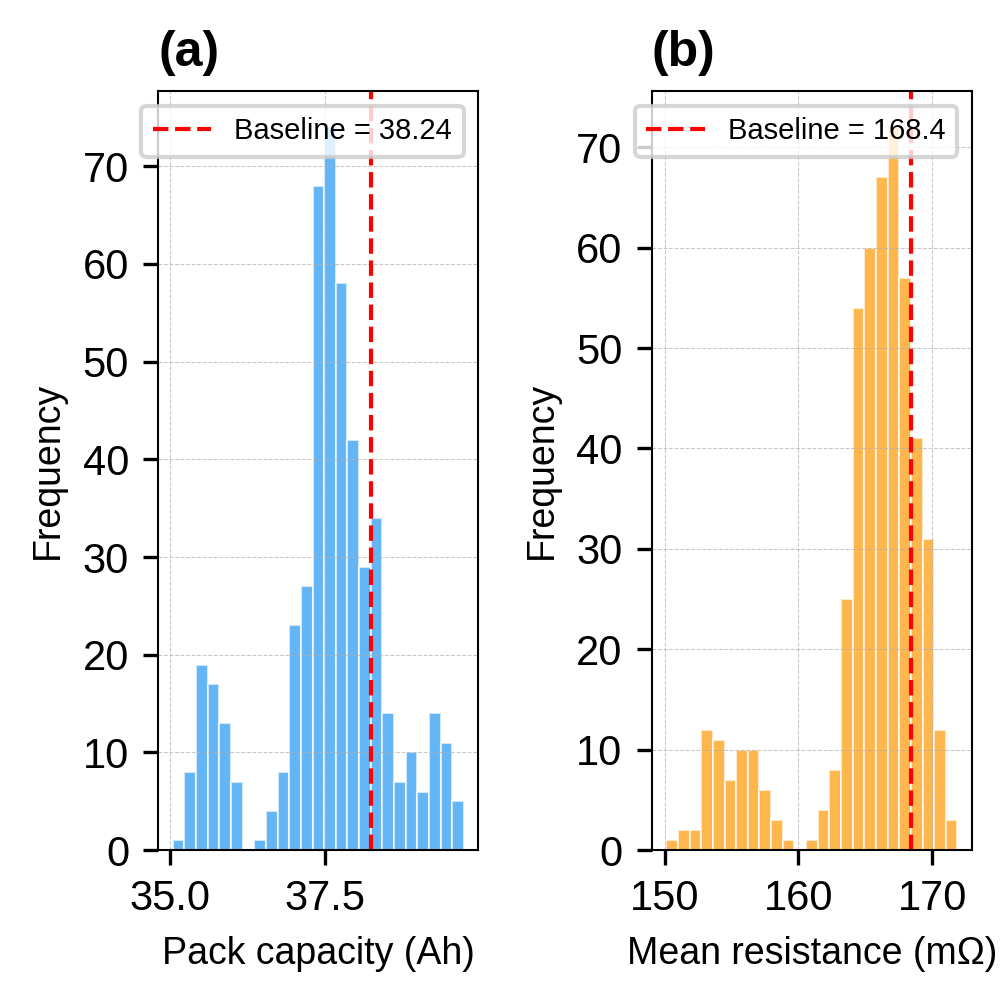

Supplement: S3 Fig — Distribution of pack capacity, resistance, and Jaccard index under bootstrap and worst-case scenarios (B = 500). (TIFF) [file pone.0353394.s010.tiff]
